# Cellular Origins of the Lymphatic Endothelium: Implications for Cancer Lymphangiogenesis

**DOI:** 10.3389/fphys.2020.577584

**Published:** 2020-09-24

**Authors:** Laura Gutierrez-Miranda, Karina Yaniv

**Affiliations:** Department of Biological Regulation, Weizmann Institute of Science, Rehovot, Israel

**Keywords:** lymphatic, origin, lymphangiogenesis, tumor, development

## Abstract

The lymphatic system plays important roles in physiological and pathological conditions. During cancer progression in particular, lymphangiogenesis can exert both positive and negative effects. While the formation of tumor associated lymphatic vessels correlates with metastatic dissemination, increased severity and poor patient prognosis, the presence of functional lymphatics is regarded as beneficial for anti-tumor immunity and cancer immunotherapy delivery. Therefore, a profound understanding of the cellular origins of tumor lymphatics and the molecular mechanisms controlling their formation is required in order to improve current strategies to control malignant spread. Data accumulated over the last decades have led to a controversy regarding the cellular sources of tumor-associated lymphatic vessels and the putative contribution of non-endothelial cells to this process. Although it is widely accepted that lymphatic endothelial cells (LECs) arise mainly from pre-existing lymphatic vessels, additional contribution from bone marrow-derived cells, myeloid precursors and terminally differentiated macrophages, has also been claimed. Here, we review recent findings describing new origins of LECs during embryonic development and discuss their relevance to cancer lymphangiogenesis.

## Structure and Functions of the Lymphatic System

The lymphatic system is composed of an extended network of vessels and secondary lymphoid organs [e.g., lymph nodes, Peyer Patches, spleen and mucosa-associated lymphoid tissue (MALT)], distributed throughout the entire organism. Lymphatic vessels are arranged in a tree-shaped hierarchy. Each vessel type – capillaries, pre-collecting and collecting lymphatics –, displays specific structural and functional features (Ulvmar and Mäkinen, 2016; Petrova and Koh, 2020). The lymphatic capillaries are blind-ended tubes formed by one single lining of lymphatic endothelial cells (LECs) that exhibit discontinuous button-like junctions to facilitate fluid entry into the vessel (Leak and Burke, 1966; Baluk et al., 2007). In contrast, collecting lymphatics are complex vessels with continuous intracellular zipper-like junctions, basement membrane and a contractile smooth muscle layer that help pump the lymph through the lymph node back to the bloodstream (Baluk et al., 2007; Muthuchamy and Zawieja, 2008). In addition, they contain bi-leaflet valves that prevent lymph backflow, and assure unidirectional transport (Kampmeier, 1928; Smith, 1949). Besides uptaking fluids and plasma solutes from the interstitium and returning them back to the venous circulation, lymphatics also actively regulate immune trafficking of antigens and antigen-presenting cells toward the lymph nodes (Alitalo and Carmeliet, 2002), and absorb lipids in the gut through the intestinal lacteals (Tso and Balint, 1986; Huang et al., 2015).

Absence or malfunction of lymphatic vessels leads to the onset of many pathologies, including lymphedema, an incurable disease characterized by a disabling swelling of the extremities, accompanied by recurrent life-threatening infections (Alitalo and Carmeliet, 2002; Brouillard et al., 2014; Yuan et al., 2019; Azhar et al., 2020). In addition, since lymphatics proliferate under inflammatory conditions (Pullinger and Florey, 1937) and are responsible for immune cell clearance and resolution of inflammation; dysfunctional lymphatics are associated with chronic inflammatory and autoimmune diseases like psoriasis (Kunstfeld et al., 2004) and rheumatoid arthritis (reviewed in Schwartz et al., 2019).

During cancer progression lymphatic vessels can play dual roles (reviewed in Oliver et al., 2020; Petrova and Koh, 2020; Vaahtomeri and Alitalo, 2020). On the one hand, they promote tumor metastasis by providing malignant cells with an escape conduit for dissemination toward the lymph nodes and distal metastatic niches. Accordingly, the presence of lymphatic metastasis is correlated with poor patient prognosis and survival (Pasquali et al., 2013; Stacker et al., 2014; Wilczak et al., 2018). On the other side, the presence of functional lymphatics boosts anti-tumoral immune response and facilitates the delivery of chemotherapy agents enhancing their effect (Lund et al., 2016; Fankhauser et al., 2017; Song et al., 2020). Hence, a profound characterization of the mechanisms underlying lymphatic formation is required in order to improve current strategies for controlling tumor progression, by preventing or encouraging lymphatic vessel growth.

## Embryonic Development of Lymphatic Vessels

It is a widely held view that during embryogenesis the first LECs derive from venous structures (Sabin, 1902; Wigle and Oliver, 1999; Yaniv et al., 2006; Srinivasan et al., 2007). Specifically, at E9.5 of mouse development, a subpopulation of cells within the cardinal veins acquires lymphatic identity (You et al., 2005; François et al., 2008; Srinivasan et al., 2010) by inducing the expression of prospero homeobox protein 1 (PROX1) the main driver of lymphatic commitment and differentiation (Wigle and Oliver, 1999; Wigle, 2002; Srinivasan and Oliver, 2011). Between E9.5 until E14.5, PROX1 positive cells upregulate the expression of additional lymphatic-specific markers like the membrane receptors vascular endothelial growth factor 3 (VEGFR3) (Kaipainen et al., 1995), Lymphatic vessel endothelial hyaluronan receptor 1 (LYVE1) (Banerji et al., 1999), and podoplanin (PDPN) (Breiteneder-Geleff et al., 1999). Then, following secretion and processing of vascular endothelial growth factor C (VEGFC), VEGFR3+ responsive LECs sprout out of the veins and migrate toward the signal source to assemble the primitive lymphatic structures, the lymph sacs (Wigle and Oliver, 1999; Wigle, 2002). Subsequent sprouting and remodeling of the lymph sacs help assemble a mature lymphatic plexus.

**FIGURE 1:**
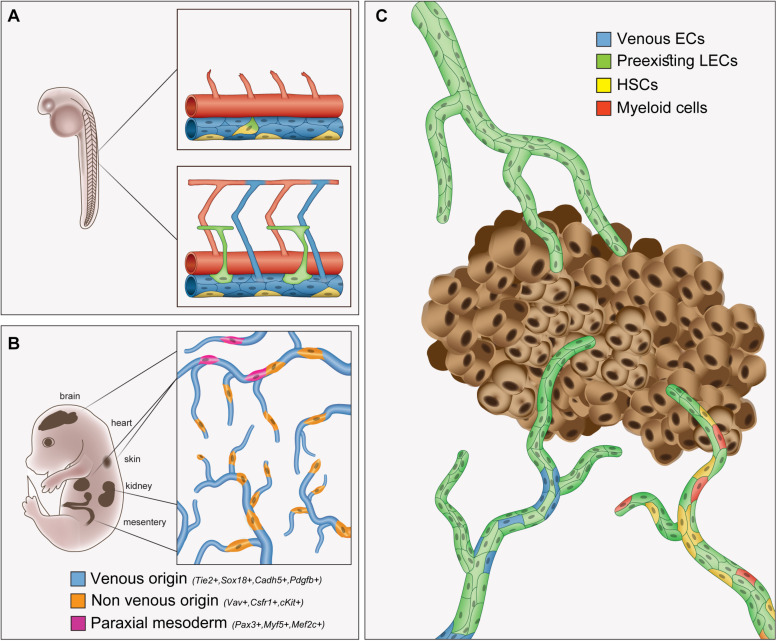
Overview of the origins of lymphatic endothelial cells in embryonic development and cancer. Schematic illustrations depicting the origins of cells contributing to the formation of **(A)** early lymphatics in the zebrafish trunk, **(B)** organ-specific lymphatics in the mouse embryo and **(C)** tumor-associated lymphatics. **(A)** A cluster of angioblasts (yellow) located in the ventral side of the PCV are the source of the parachordal cells (green), the primordial lymphatic progenitors in the embryonic zebrafish trunk. Arteries are depicted in red and veins in blue. **(B)** Lineage-tracing experiments in mice revealed mixed origin of organ-specific lymphatic networks. Venous (blue), paraxial mesoderm (pink) and additional non-venous (orange) origins give rise to organ-specific LECs (detailed in Table1). **(C)** Distinct cell populations contribute to tumor lymphatics. Tumor LECs form primarily via sprouting lymphangiogenesis from pre-exiting lymphatics (green). In addition, BMDCs, including hematopoietic stem cells (HSCs), and cells of the myeloid lineage have also been identified as non-endothelial sources of tumor LECs.

A similar process was found to take place in aquatic animals. In zebrafish, a cluster of angioblasts in the floor of the posterior cardinal vein (PCV) was shown to give rise to lymphatic progenitors in the developing embryo (Nicenboim et al., 2015). The cells committed to the lymphatic fate, migrate to the dorsal part of the PCV and bud toward the midline of the embryo to form the primordial parachordal cells (also known as parachordal lymphangioblasts or PLs), the building blocks of the lymphatic system (Küchler et al., 2006; Yaniv et al., 2006). Another study revealed the existence of bipotent Prox1+ precursor cells in the PCV that generate lymphatics through asymmetric cell division (Koltowska et al., 2015) (Figure 1A). Although the process of lymphatic formation is highly conserved among vertebrates, certain divergences between the mammalian and zebrafish systems have also been described (Tao et al., 2011; van Impel et al., 2014; Semo et al., 2016).

Once differentiated, LECs respond to two major stimuli: VEGF-C (Joukov et al., 1996) and VEGF-D (Achen et al., 1998), both of them VEGFR3 ligands. VEGF-C controls both proliferation and migration of LEC (Joukov et al., 1996; Jeltsch, 1997). The VEGF-C/VEGFR3 axis is very active during early lymphangiogenesis in mouse embryonic development (Kukk et al., 1996; Veikkola et al., 2001) and dysregulation of either of these factors results in lymphatic malformations (Dumont, 1998; Karkkainen et al., 2000, 2004). Although VEGF-C does not control LEC specification *per se*, its function is indispensable for the sprouting of primordial lymphatics from the cardinal vein in mice and zebrafish (Karkkainen et al., 2004; Shin et al., 2016). Accordingly, *Vegfc*^–/–^ mouse embryos lack primordial lymphatic structures, develop edema and die prematurely. In addition, loss of *Vegfc* expression or absence of VEGF-C in mice results in the development of defective lymphatics and lymphedema (Mäkinen et al., 2001; Karkkainen et al., 2004). In similar fashion, zebrafish *vegfc* mutants are devoid of lymphatic vessels (Villefranc et al., 2013; Le Guen et al., 2014) and heterozygous *vegfc^+/–^* animals fail to establish proper lymphatic vessels in various organs, including the heart (Gancz et al., 2019b). Both VEGF-C and VEGF-D are produced and secreted as pre-propeptides and they require to undergo a proteolytic cleavage in order to acquire their mature and active form (Joukov et al., 1997; Stacker et al., 1999). VEGF-C processing requires the formation of the VEGFC-CCBE1-ADAMTS3 multicomplex (Jeltsch et al., 2014) and loss of any of this components leads to lymphatic defects. Collagen and calcium binding EGF domains 1 (CCBE1) is a secreted regulator of VEGF-C activation necessary for proper lymphatic vessel formation in mammals (Bos et al., 2011; Hägerling et al., 2013; Roukens et al., 2015) and zebrafish (Hogan et al., 2009; Le Guen et al., 2014). In addition, *CCBE1* mutations were identified in patients with several lymphatic vessel malformations (Alders et al., 2009; Connell et al., 2010). A disintegrin and metalloproteinase with thrombospondin motifs 3 (ADAMTS3) is a VEGF-C protease, whose total deletion (i.e., *Adamts3*^–/–^ mice) mirrored the phenotypes observed in *Ccbe1* and *Vegfc* null embryos (Bui et al., 2016). Likewise, loss of the Vegfc-processing components *adamts3* and *adamts14* in zebrafish also mimicked the *vegfc* mutant phenotype exhibiting absent trunk, facial and meningeal lining lymphatics (Wang et al., 2020).

VEGFR3 is initially expressed in all embryonic ECs but later on becomes restricted to the LECs (Kaipainen et al., 1995) and fenestrated endothelial cells (Partanen et al., 2000). *Vegfr3* null mutant mice die at midgestation due to delayed vascular development and cardiovascular defects (Dumont, 1998). Likewise, zebrafish *flt4* mutants lack early lymphatic vessels (Kok et al., 2015) as well as adult cardiac lymphatics (Gancz et al., 2019b).

Notably, during the past two decades specific mutations on *VEGFC* (Gordon et al., 2013; Balboa-Beltran et al., 2014; Fastré et al., 2018; Nadarajah et al., 2018), *VEGFR3* (Ferrell, 1998; Irrthum et al., 2000; Karkkainen et al., 2000; Evans, 2003; Brice, 2005; Daniel-Spiegel et al., 2005; Mizuno et al., 2005; Butler et al., 2007; Ghalamkarpour et al., 2006, 2009; Dai et al., 2018) and additional lymphangiogenesis-related genes, have been found to be strongly associated to the onset of primary lymphedema (reviewed in Oliver et al., 2020).

## The Origins of Lymphatics Endothelial Cells

The earliest description of the lymphatic system dates back to Hippocrates, but it was not until the early 1600, where observations of nodes and ducts carrying milky liquid were reported by Gasparo Aselli. During the 20th century, the origin of these vessels was discussed by two conflicting theories. In 1902, the anatomist Florence Sabin suggested, based on her studies of lymphatic development in pig embryos, that the first lymphatic ducts originated from veins (Sabin, 1902). Contrary to this idea, the anatomists George S. Huntington and Charles F. W. McClure postulated that the jugular lymph sacs develop from a cluster of mesenchymal cells in cat embryos (Huntington and McClure, 1910). During the last century, several studies supporting one view or the other were published, underscoring the active debate surrounding the field. While few reports analyzing lymphatic development in turtle (van der Jagt, 1932), chick and quail (Schneider et al., 1999; Wilting et al., 2006) and *Xenopus* (Ny et al., 2005) postulated a dual origin for lymphatic vessels (i.e., with contribution of both the mesenchymal and the venous compartments) (reviewed in Semo et al., 2016); the model claiming a sole venous origin for the lymphatic endothelium became predominant (Lewis, 1905; Wigle and Oliver, 1999; Wigle, 2002; Yaniv et al., 2006; Srinivasan et al., 2007).

Most recently, however, several studies have revived this debate by revealing new unexpected origins for LECs both during early development and in certain organs (reviewed in Semo et al., 2016; Ulvmar and Mäkinen, 2016; Petrova and Koh, 2018; Gancz et al., 2019a). Accordingly, while the majority of organ-specific lymphatics appears to form through sprouting lymphangiogenesis from neighbor lymphatic vessels, the presence of LECs lineage-traced to different origins was detected. Importantly, the nature of the specific progenitors from which LECs can stem varies in different organs, suggesting that the origin of these LEC populations might be contingent to the specific tissue environments and driven by organ-specific signals (Figure 1B). The cellular origins of distinct lymphatic populations identified so far are summarized in Table1.

**TABLE 1:** Cellular origins of LECs during embryonic development.

Lymphatics	Species	Venous source	Non-venous source
Jugular sacs	Pig embryo	Sabin (1902)	
Jugular sacs	Cat embryo		Mesenchymal cells (Huntington and McClure, 1910)
Jugular sacs	Mouse embryo	*Tie2+* (Wigle and Oliver, 1999; Wigle, 2002; Srinivasan et al., 2007)	
Parachordal cells	Zebrafish embryo	*fli1a+* PCV (Yaniv et al., 2006)	*flt1_9a+* angioblasts (Nicenboim et al., 2015)
Facial lymphatics	Zebrafish embryo	*lyve1b+* CCV and PHS (Okuda et al., 2012; Eng et al., 2019)	VA-L (Eng et al., 2019)
Dermal lymphatics	Mouse embryo	*Tie2+* cervical/thoracic region (Martinez-Corral et al., 2015) *Sox18+/Cadh5+/Tie2*+ cervical/thoracic region (Pichol-Thievend et al., 2018)	*Tie2-/Vav-* dorsal/midline/lumbar region (Martinez-Corral et al., 2015) *Pax3+* thoracic/lumbar/sacral region (Stone and Stainier, 2019) *Myf5+* Ear skin (Stone and Stainier, 2019) *Mef2c+* cervical/thoracic region (Stone and Stainier, 2019)
Cardiac lymphatics	Mouse embryo	*Tie2+* (Klotz et al., 2015)	*Vav1+/Pdgfb+/Csfr1+* (Klotz et al., 2015) *Apln-* and *Apj-* (Gancz et al., 2019b) *Isl1+* (Maruyama et al., 2019; Lioux et al., 2020) *Pax3+* (Stone and Stainier, 2019)
	Zebrafish	*lyve1b+/prox1a+/mrc1a+/flt4+* (Gancz et al., 2019b)	Isolated LECs: Undetermined (Gancz et al., 2019b)
Mesenteric lymphatics	Mouse embryo	*Pdgfb+* (Stanczuk et al., 2015)	*cKit+/Vav1-* (Stanczuk et al., 2015)
Lymph nodes LECs	Mouse embryo		*Nestin+* (Koning et al., 2016)
Renal lymphatics	Mouse embryo	*Tie2+* ascending vasa recta: (Kenig-Kozlovsky et al., 2018)	Isolated LECS: Undetermined (Jafree et al., 2019)
Brain	Mouse embryo	Undetermined Aspelund et al. (2015), Louveau et al. (2015)	Undetermined Aspelund et al. (2015), Louveau et al. (2015) *Myf5+* (Stone and Stainier, 2019)
	Zebrafish	*mrc1a*+ meningeal lymphatics (Castranova et al., 2020) *mrc1a+* mural LECs (muLECs) */flt4+/prox1a+/lyve1b+* (Bower et al., 2017; Galanternik et al., 2017; van Lessen et al., 2017)	
Schlemm’s canal	Mouse embryo	*Kdr+* lumbar vasculature (Kizhatil et al., 2014)	

One of the first such examples was the superficial dermal lymphatic network, for which two different LEC origins were reported (Martinez-Corral et al., 2015; Pichol-Thievend et al., 2018). Martinez-Corral et al. (2015) claimed that skin lymphatics in the cervical and thoracic regions of the mouse embryo, form via transdifferentiation of *Tie2*-expressing venous structures and subsequent sprouting from the jugular lymph sacs. In contrast, lymphatics in the lumbar and dorsal midline areas, arise from *Tie2-*negative and *Vav-*negative progenitors, suggesting a non-endothelial and non-hematopoietic origin (Martinez-Corral et al., 2015). Later on, Pichol-Thievend et al. (2018) provided evidence for a novel endothelial progenitor subpopulation located within the capillary bed that gives rise to murine dermal lymphatics in the cervico-thoracic region. According to this study, PROX1+ progenitors in the local vascular plexus bud off the capillaries as single LECs or as small clusters, in a *Ccbe1-*dependent manner. Subsequently, these LECs proliferate and expand, prior to merging into the preexisting dermal lymphatic vasculature (Pichol-Thievend et al., 2018).

The development of mesenteric lymphatics was also shown to involve mixed cell contribution (Mahadevan et al., 2014; Stanczuk et al., 2015). While the mesenteric lymph sacs derive from veins at the mesenteric root, the mesenteric vessels were found to originate from *Pdgfb+ cKit*+ hemogenic endothelium, independently of the definitive hematopoietic lineage *Vav-* (Stanczuk et al., 2015).

A similar dual origin – venous and non-venous – of lymphatic progenitors was found also in zebrafish. In this animal, the facial lymphatic network develops in a sequential manner through initial sprouting from the common cardinal vein (CCV) and primary head sinus (PHS), which are of venous origin, followed by an additional non-venous population of lymphangioblasts that connects to this main sprout (Okuda et al., 2012; Eng et al., 2019). In addition, a pool of specialized angioblasts was identified in the floor of the PCV that gives rise to LECs (Nicenboim et al., 2015) as well as to arterial and venous ECs (Hen et al., 2015). These angioblasts were shown to be molecularly distinct from surrounding venous cells, displaying enriched expression of angioblast and arterial markers, and to arise directly from a restricted population in the lateral plate mesoderm.

During the past years, an increasing number of lineage-tracing based studies has determined also the heterogeneous ontology of cardiac lymphatics. In mouse embryos, venous derived lymphatics were shown to give rise to the vessels paralleling the coronaries, while non-venous, *Tie2*-negative yolk sac derived progenitors (Klotz et al., 2015) contribute to the formation of PROX1+ LECs in the developing heart. Recently, three independent studies (Gancz et al., 2019b; Harrison et al., 2019; Vivien et al., 2019) described the development of cardiac lymphatics in zebrafish as well as their role during cardiac regeneration. Interestingly, in addition to conventional lymphatic vessels spanning the cardiac ventricle, Gancz et al. (2019b) reported the presence of isolated *prox1+, lyve1b+, mrc1+ and flt4+* LEC clusters in the zebrafish heart that developed initially into single capillaries, and later on connected to the existing lymphatic plexus. Moreover, a similar PROX1+ population of isolated LEC clusters was detected in the hearts of E14.5 mouse embryos (Gancz et al., 2019b), suggesting that the mechanisms underlying cardiac lymphatic formation are conserved across vertebrates (Gancz et al., 2019b). The second heart field (SHF), a multipotent cell population that contributes to several cardiac structures (Buckingham, 2016; Meilhac and Buckingham, 2018), has also been proposed as an additional non-venous source of cardiac LECs (Maruyama et al., 2019; Lioux et al., 2020). Specifically, *Isl1+* pharyngeal mesoderm-derived progenitors were shown to contribute to the formation of lymphatics in the outflow tract and in the ventral ventricles of mouse embryos (Maruyama et al., 2019). Further clonal studies and lineage tracing experiments showed that *Islet1+* and *Mef2c+* SHF precursors contributed to the formation of cardiac ventral lymphatics but not supported the formation of the dorsal lymphatics. Furthermore, they confirmed that a specific subset of *Isl1+* sub-mesothelial cells give rise to PROX1+/LYVE1+ vascular structures that comprise around 50% of ventral LECs (Lioux et al., 2020).

The kidney provides another example of how different LEC populations assemble to generate an organ-specific lymphatic plexus. Mouse and human kidneys were reported to bear one “traditional” network of lymphatic capillaries that forms from a ring-like anastomosis in the renal hilum, and a separate population of PROX1+/LYVE1+ isolated LEC clusters similar to those described in the heart (Gancz et al., 2019b), suggesting that both lymphangiogenesis and lymphvasculogenesis play a role in renal lymphatic development (Jafree et al., 2019). Although the isolated LECs were assumed to stem from non-endothelial structures, based on their low PECAM and endomucin expression, the identity of the specific progenitors was not determined by lineage-tracing experiments.

Lymph node LECs were suggested to derive from *Nestin+* precursors. Fate mapping of these cells in mouse embryos revealed that they contribute to the formation of both CD31+ endothelial cells, and CD31- mesenchymal stromal niches in the lymph nodes. During postnatal development though, *Nestin* expression becomes predominant in the endothelial compartment including the capillaries, high endothelial venules and LECs (Koning et al., 2016). Nevertheless, given that Nestin expression is detected in a wide variety of cells, such as neural stem cells (Mignone et al., 2004) and stem cells from the mesenchymal lineage (Méndez-Ferrer et al., 2010) including endothelial cells (reviewed in Bernal and Arranz, 2018), additional experiments will be required in order to verify the exact origins of lymph node LECs.

Although the earliest observations of meningeal lymphatics date from investigations of Paolo Mascagni in the 18th century, they were mostly neglected (reviewed in Da Mesquita et al., 2018a). Lately, however, two elegant studies have described the presence of a lymphatic network in the mammalian meninges, challenging the old dogma that considered the brain as an immune-privileged organ (Aspelund et al., 2015; Louveau et al., 2015). Louveau et al. (2015) described the presence of meningeal lymphatic vessels aligned with the dural sinuses. These peri-sinusal lymphatics expressed the classical LEC markers such as LYVE1, PDPN, and PROX1. Importantly, a similar LYVE1+, PDPN+, and CD68- structure was also identified in the human dura. These meningeal lymphatic vessels extend from the eye and olfactory bulb toward the sinuses, exiting the skull along vein, arteries and cranial nerves (Aspelund et al., 2015). During the past few years several studies have demonstrated the role of meningeal lymphatics in maintaining brain homeostasis, by clearing fluids, macromolecules and immune cells from the cerebrospinal and interstitial fluids, into the cervical nodes (Ma et al., 2017; Da Mesquita et al., 2018b; Louveau et al., 2018; Ahn et al., 2019). The discovery that this drainage function appears to decrease in aged mice, has prompted research into the putative role of lymphatics in neurodegenerative disorders (Ma et al., 2017; Da Mesquita et al., 2018b; Ahn et al., 2019; Wang et al., 2019; Zou et al., 2019). Hence, regulating the meningeal lymphatics function should be further studied as a therapeutic approach to delay the initiation or development of several neurological pathologies.

Interestingly, a population of isolated, non-lumenized LECs, expressing all the LEC hallmarks was recently identified in the zebrafish brain (Bower et al., 2017; Galanternik et al., 2017; van Lessen et al., 2017). Anatomically, these perivascular LECs were shown to sprout from the choroidal vascular plexus and to cover most of the adult brain (Galanternik et al., 2017; van Lessen et al., 2017). Similar to perivascular macrophages, Mato cells (Mato et al., 1984) or brain perivascular fluorescent granular perithelial cells (FGPs), this population of perivascular mural LECs (muLECs) display high endocytic and phagocytic capabilities (Bower et al., 2017; Galanternik et al., 2017; van Lessen et al., 2017). In addition, it has been proposed that these muLECs might promote or support vessel formation by secreting angiogenic and lymphagiogenic growth factors during embryonic development (Bower et al., 2017) and vascular regeneration (Chen et al., 2019). As reported by Chen et al. (2019), muLECs could penetrate into the brain parenchyma upon cerebrovascular damage in order to resolve edema and guide the formation of new vessels into the injured tissue. Aside from muLECs, which are detected throughout the brain, proper meningeal lymphatics were also detected in *larvae* and adult zebrafish. *Mrc1+* meningeal vessels were shown to originate via sprouting of the facial lymphatics and were suggested to play a role in immune cell trafficking (Castranova et al., 2020).

The eye was as well thought to lack lymphatic vessels, until it was discovered that the Schlemm’s canal – the vessel that regulates intraocular pressure via drainage of the aqueous humor from the eye chamber –, develops as a hybrid blood and lymphatic structure, through “canalogenesis.” This process initiates with the sprouting of ECs from the limbal vascular plexus and the radial vessels, into the corneal intermediate zone, and continues with the clustering of tip cells from the aforementioned blood vessels in the limbus, and assembly of a primordial chain of cells. The developing structure further extends and matures to form a lumenized vessel with distinct properties in its external and internal walls (Kizhatil et al., 2014). Although the Schlemm’s canal derives from blood ECs and expresses most of the common blood EC markers, it is considered an hybrid vessel, as the cells in the inner wall express *Prox1* and VEGFR3 although at lower levels than regular lymphatics (Kizhatil et al., 2014; Truong et al., 2014). Another specialized hybrid structure can be found in the ascending vasa recta in the kidney medulla, a structure devoid of *bona fide* lymphatics. The ascending vasa recta (AVS) is a fenestrated vessel that exhibits both lymphatic and blood venous features and is essential for proper fluid drainage. Lineage tracing experiments revealed that ASV as most of the renal microvasculature derived from *Tie2*-expressing cells (Kenig-Kozlovsky et al., 2018).

Besides organ-specific lymphatics, recent lineage-tracing experiments in mice using the dermomyotome *Pax3* and *Myf5* and the SHF *Mef2c* specific drivers revealed that lymphatic progenitors can be traced back to the paraxial mesoderm, suggesting a common origin shared between LECs and the skeletal muscle lineage (Stone and Stainier, 2019). Analysis of *Pax3-*Cre+ cells showed that PROX1-expressing LEC precursors were present in the dorso- lateral wall of the mouse CV, and subsequently in the jugular lymph sacs. Additionally, *Pax3+* cells gave rise to lymphatics in the skin, the liver and the cardiopulmonary system. In similar fashion, *Myf5+* muscle progenitors were shown to contribute to the formation of meningeal and lymphatic capillaries in the ear skin, and *Mef2c+* cells were found in the jugular lymph sacs, heart and skin lymphatics (Stone and Stainier, 2019).

As a whole, work in the recent years has begun to shed light on the heterogeneous origins of organ-specific lymphatics. Yet, it is important to keep in mind that while lineage tracing approaches have been instrumental for identifying novel progenitor populations and establishing lineage relationships within cell progeny/populations (Das and Yaniv, 2020), this technique harbors also intrinsic technical limitations. Unspecific or leaky drivers might erroneously label undesired populations, whereas the absence of specific labeling in certain cells can derive from an inefficient driver, all of these leading to potential misinterpretation of the results (reviewed in Semo et al., 2016). These limitations become especially relevant when coming to analyze the potential contribution of venous/endothelial vs. non-endothelial cells to the formation of tumor-associated lymphatic vessels.

## Formation of Cancer-Associated Lymphatics

As part of the tumor microenvironment, the endothelial cells maintain a coordinated crosstalk with the tumor cells. As the diffusion of oxygen within the tissue decreases (Thomlinson and Gray, 1955; Goldacre and Sylvén, 1962); the tumor cells secrete growth factors that induce the formation of new vessels, allowing them to obtain the nutrients and oxygen required for their survival (Lugano et al., 2020). Since the initial observations of the tumor vasculature in the early 1900s, it was quite clear that the tumor-associated vasculature forms a heterogeneous network (Borst, 1902; Goldmann, 1908; De Palma et al., 2017). Tumor ECs exhibit significant morphological and functional differences when compared to their normal counterparts (Croix, 2000). In addition, they harbor cytogenetic alterations (Hida et al., 2004) and high cell turnover (Hobson and Denekamp, 1984). Nowadays it is widely accepted that the tumor vasculature is organized in a non-hierarchical manner and consists of leaky vessels (Hashizume et al., 2000; Morikawa et al., 2002) with irregular patterning and flow (Jain, 1988).

Once the solid tumor becomes vascularized, there is a rapid switch from a dormant to a malignant state that promotes further growth and tissue dissemination (Lugano et al., 2020). In addition to blood ECs, lymphatic vessels are also present in the tumor microenvironment, and are considered to play an active role in primary tumor progression, metastatic spread and immunomodulation (Stacker et al., 2014; Garnier et al., 2019; Oliver et al., 2020; Petrova and Koh, 2020; Vaahtomeri and Alitalo, 2020). On the one hand, tumor-associated lymphatics uptake the interstitial fluid containing cells and macromolecules of tumor mass origin, and drain them to the sentinel lymph node. The lymphatic vessels thus act as a circulation conduit for the tumor cells to escape the primary site and facilitate cell dissemination to distal organs. Accordingly, lymphatic tumor coverage correlate with poor patient prognosis (Dadras et al., 2003; Pasquali et al., 2013; Stacker et al., 2014; Wilczak et al., 2018). On the other hand, lymphatic vessels carry the antigens and antigen-presenting cells from the periphery to the lymph nodes to initiate the anti-tumor immunity and activate T cell response. LECs were shown to recruit dendritic cells via CCL21-CCR7 signaling, that migrate to present specific tumor antigens to the T cells in the lymph nodes. Therefore, dysfunctional lymphatics hamper a proper immune activation and response (Garnier et al., 2019).

The tumor and tumor microenvironment cells secrete a plethora of lymphangiogenic growth factors that promote lymphatic vessel formation (Vaahtomeri et al., 2017). Among them, the secretion of VEGF-C and VEGF-D stimulate the formation, proliferation and sprouting of LECs (Joukov et al., 1996; Jeltsch, 1997; Veikkola et al., 2001; Hirakawa et al., 2007) attracting them into the peri- and intra-tumoral areas. The newly formed lymphatics are detected within the tumor mass and in its periphery in close contact with the tumor stroma. While, intratumoral lymphatics are for the most part not functional due to pressure collapse (Leu et al., 2000; Padera, 2002), the peritumoral ones are functional, but appear inflamed and dilated (Leu et al., 2000; Skobe et al., 2001a; Isaka et al., 2004). The formation of lymphatic vessels is not restricted to the primary tumor since the sentinel draining lymph node also forms new lymphatic vessels in preparation of the metastatic niche (Hirakawa et al., 2005, 2007; Stacker et al., 2014).

The active VEGF-C/VEGF-D/VEGFR3 signaling leads to increased lymphatic vessel permeability and intratumoral pressure, thereby augmenting the outflow toward the lymph node and facilitating the intravasation of cancer cells. It is though that the increased drainage activity of the interstitial fluid toward the lymphatic vasculature and lymph nodes significantly contributes to the lymphatic metastatic spread. Indeed, VEGF-C, VEGF-D and VEGFR3 levels are correlated with an increased incidence of lymph node and distal metastasis (Skobe et al., 2001a,b; Stacker et al., 2001; He et al., 2005; Hoshida et al., 2006; Hirakawa et al., 2007; Su et al., 2006, 2007; Karnezis et al., 2012).

## The Cellular Origins of Cancer Lymphatics

In contrast to the wealth of information describing the formation of tumor-associated blood vessels, relatively little is known about the process of tumor-induced lymphangiogenesis. Especially, the cellular origins of newly formed lymphatic vessels have been a matter of active debate during the past decade. While early reports suggested that tumor lymphatic vessels derive solely from pre-existing lymphatics (He et al., 2004), other investigations proposed alternative origins for tumor-LECs (Religa et al., 2005; Zumsteg et al., 2009). One of the first studies addressing this question made use of GFP-tagged bone marrow (BM) cell transplantation into mice bearing Lewis lung carcinoma (LLC) or B16 melanoma subcutaneous tumors. Assessment of the newly formed lymphatics surrounding the tumors revealed that although GFP+ cells were found in close proximity to the vessels, no double GFP+ (BM-derived cells, BMDCs) and LYVE1+ LECs were detected, suggesting that peritumoral lymphatics arise primarily from pre-existing vessels, with very little, if any, BDMC contribution (He et al., 2004). In contrast, the BM-derived GFP+ cells were markedly detected in the vicinity of new lymphatic vessels after tumor implantation, suggesting a potential role in neo-lymphangiogenesis through paracrine effects on existing lymphatic vessels. In addition, He et al. (2005) demonstrated that implantation of human LNM35 lung cancer cells expressing high levels of VEGF-C, robustly induced lymphatic sprouting from LYVE1+ vessels in an ear model, further supporting a lymphatic vessel origin for newly formed lymphatic capillaries.

In contrast to these results, other studies have shown integration of certain types of BMDCs into lymphatic vessels during pathological conditions, including tumor-induced lymphangiogenesis (reviewed by Park et al., 2011; Ran and Volk-Draper, 2020). Religa et al. (2005) for instance, showed that circulating BDMCs generate lymphatic vessels *in vivo* in GFP+ BM transplanted mice. In this study, GFP+;LYVE1+ cells were found in the cornea and in the enveloping lymphatics of T241 tumors, suggesting that circulating BM progenitors can indeed be incorporated into new lymphatic vessels (Religa et al., 2005). These progenitor cells were described to incorporate into LECs from many tissues and remain for rather long periods of time, although it was shown that they only comprise a minimum portion of the vasculature in both normal and tumor-associated vasculature (Jiang et al., 2008).

Similarly, a small cluster (∼8%) of cultured BM-derived mononucleated cells that upregulated PDPN expression, was shown to incorporate into lymphatic vessels of the cornea, skin, ear wounds and tumors, following B16-F1 melanoma injection. In addition, this population of PDPN+ lymphatic endothelial progenitors, was significantly enlarged in the bone marrow and peripheral blood of mice harboring tumors, suggesting that these cells could be activated and mobilized during tumorigenesis (Lee et al., 2010).

While these and other early studies (Maruyama et al., 2005; Jiang et al., 2008; Attout et al., 2009; Yamashita et al., 2009; Hall et al., 2012; Ran and Montgomery, 2012) supported the putative contribution of hematopoietic stem cells (HSCs) to neo-lymphangiogenesis in pathological settings; it is important to bear in mind that the expression of PROX1 in the integrated cells was not analyzed, and that the LEC phenotype was confirmed based only on the expression of markers such as PDPN, LYVE1, and VEGFR3, which are expressed by both LECs and hematopoietic cells including monocytes and macrophages.

Additional reports, focusing specifically on the contribution of BMDCs of the myeloid lineage to tumor lymphangiogenesis, examined also PROX1 expression (Zumsteg et al., 2009; Volk-Draper et al., 2017, 2019). Zumsteg et al. (2009) made use of BM transfer in combination with genetic lineage tracing, to investigate the integration of myeloid cells into tumor lymphatic vessels, in both the genetic Rip1Tag2 model of pancreatic cancer, and TRAMP-C1 prostate cancer transplanted animals. Analysis of the presence of GFP+ BMDCs in the vasculature of these two mouse models revealed a minor contribution of BMDCs to the tumor lymphatic network. Interestingly, only ∼3% of the GFP+ cells co-expressed the macrophage marker F4/80 along with PROX1, LYVE1 and PDPN, suggesting the possible differentiation of macrophages toward a LEC fate. However, since not all of the integrated BMDCs expressed F4/80, this suggested mechanism remained inconclusive. The cell transplantation results were further supported by lineage tracing experiments using the CD11b reporter mice. In this case, labeled cells were found to integrate into lymphatic vessels of subcutaneously transplanted TRAMP-C1 tumors, and to co-express LYVE1 and PROX1 (Zumsteg et al., 2009).

Recently, it has been proposed that the myeloid-to-lymphatic transition could be regulated via the Toll-Like Receptor 4 (TLR4). Treatment of primary CD14+ human monocytes and CD11b+ mouse myeloid cells, with the TLR4-ligands LPS, HMGB1 and nab-PXL, triggered the upregulation of *VEGFR3*, *LYVE1* and *PDPN* transcripts, shifting the cells to a LEC-like phenotype. Furthermore, *in vitro* TLR4- reprogrammed myeloid lymphatic endothelial cell progenitors (M-LECPs), generated functional cells that integrated into LYVE1+ tumor lymphatics in murine breast cancer models (Volk-Draper et al., 2017). A follow-up study suggested that the tumors might boost the release of myeloid precursors, since CD14+ monocytes expressing LEC specific markers were abundant, albeit being nearly absent in healthy donors. In addition, M-LECPs expressing high levels of *LYVE1, PDPN, PROX1* and *VEGFR3* were uniquely detected in the lymph vessels of breast cancer tissue and their density correlated with the development of lymphatic metastasis in breast cancer patients (Volk-Draper et al., 2019).

Contrasting results regarding the contribution of the myeloid lineage to tumor lymphatics were obtained using a *LysM:Cre* lineage tracing approach (Gordon et al., 2010). According to this study, while lineage-labeled cells integrated within tumor-related lymphatic vessels following subcutaneous implantation of either LLC or EL4 lymphoma cells, they did not express PROX1, thus arguing against a possible mechanism of differentiation of macrophages to LECs. The discrepancy between the various studies could stem from the use of different promoters driving Cre activation. Alternatively, it is possible that distinct cytokine profiles of the tumor models utilize, induce recruitment and/or differentiation of specific myeloid subpopulations into LECs.

Inflammation also takes place during tumor growth and progression. In this regard, formation of lymphatics vessels from BMDCs has also been described in inflammation models (Maruyama et al., 2005; Kerjaschki et al., 2006; Lee et al., 2010; Hos and Cursiefen, 2014). Maruyama et al. (2005) for instance, identified double positive CD11b+/LYVE1+ and CD11b+/PROX1+ cell patches in the lymphatic neo-vasculature, 3 days after corneal transplantation. These results suggested that CD11b+ macrophages, most likely originating in the bone marrow, were the source of LECs in the inflamed mouse cornea. Moreover, the formation of LECs was suppressed upon systemic depletion of macrophages by clodronate liposomes treatment, further supporting these results (Maruyama et al., 2005). Similar findings were obtained in a study analyzing human gender-mismatched transplanted kidneys. There, the rejected kidneys incorporated host-derived macrophages that differentiated into LECs, further supporting the idea that specific lymphatic endothelial precursor cells, most probably of the macrophage lineage, intervene in the formation of new lymphatics (Kerjaschki et al., 2006).

Overall, the mechanisms regulating the formation of tumor-associated lymphatics and, in particular, the putative contribution from non-endothelial and bone marrow-derived cells, still remain controversial (Figure 1C). Especially, the observed differences in the precursor type, their ability to be mobilized and incorporated into lymphatic vessels and whether a defined LEC fate is ultimately acquired, varies significantly among the different studies. It is important to bear in mind that there are many variables in the tumor models, starting from the tumor type, their aggressiveness and localization and their metastatic potential. Moreover, there are differences within the mouse models utilized, including genetic background, preconditions, treatments, etc. Since different cell types have been described to give rise to organ-specific lymphatic vessels during embryonic development, it would be interesting to investigate for instance, whether BMDCs are differentially activated in tumors of different organs.

Another question that remains open is the potential mechanism by which circulating cells integrate into lymphatic vessels. The contribution of HSCs and myeloid cells to tumor lymphangiogenesis was typically inspected several weeks after bone marrow transplantation or adoptive transfer of particular populations, thus precluding the understanding of cell differentiation and recruitment events that may occur at early stages of the process. Consequently, it remains to be determined whether the naïve stem-like cells or myeloid cells, which were shown to contribute to lymphatic vessels (Jiang et al., 2008; Zumsteg et al., 2009; Lee et al., 2010), integrate directly into the growing lymphatics and acquire an LEC phenotype only at later stages, or contribute to lymphangiogenesis as fully differentiated LECs, through a process of lymph-vasculogenesis. Finally, an alternative scenario for lymphatic vessel formation is that clusters of vascular progenitors that are spread along the tumor tissue differentiate *in situ* into LECs and contribute to lymphatic vessel growth without prior integration into the blood vasculature, as has been shown during the formation of mesenteric lymphatic vessels in developing mouse embryos (Stanczuk et al., 2015). While there is yet no evidence supporting this model, it is possible that such events are restricted to early stages of tumor lymphangiogenesis that are rather overlooked in studies on non-EC contribution to lymphatic vessels. Intravital imaging of mice carrying both blood and lymphatic EC reporters at high spatiotemporal resolution may reveal the sequence of events and cellular transitions leading to the formation of the tumor-associated lymphatic network.

Finally, the functional relevance of non-endothelial cell contribution to tumor lymphangiogenesis, and its role on tumor progression and metastatic spread remains unclear. Lee et al. (2010) could show that implantation of bone marrow derived PDPN+ cells in the periphery of melanoma tumors in mice led to an increase in the density of VEGFR3+ vessels in the peritumoral tissues. Similarly, bone marrow derived GFP+/CD11+/PDPN+ cells injected into mice bearing EMT6 orthotopic breast carcinomas, augmented as well lymphatic vessel density (Volk-Draper et al., 2017). In a different study, Volk-Draper et al. (2019) showed that *in vitro* TLR4-mediated differentiated M-LECP inoculated in different breast cancer mice models were able to increase LYVE1+ lymphatic density, thereby resulting in a substantial increment in lymphatic metastasis. Moreover, other studies have reported the effect of blocking of myeloid cells in tumor lymphatic vasculature. Inhibition of bone marrow myeloid cell recruitment abolished M-LECPs integration into the lymphatic vessels and reduced tumor lymphatic vessel density (Volk-Draper et al., 2019). Systemic depletion of macrophages in an orthotopic urinary bladder cancer (OUBC) model also caused a decrease in tumor LYVE1+ lymphatic vessel density (Yang et al., 2011).

To conclude, a growing body of literature has examined the potential contribution of different cell types to cancer-associated lymphatics. Nevertheless, several important questions remain unanswered. For instance, the specific functions that lymphatics play at different stages of tumor progression and the molecular changes LECs may undergo between the pre-metastatic and metastatic states, as well as during therapy require further investigation. Understanding the molecular differences in LECs would undoubtedly help to design novel drugs to selectively block or activate different lymphatic subsets. Finally, further studies will need to be performed to establish the use of lymphatics density or specific lymphatic markers as prognostic markers for segregation of patients for several tumors.

## Therapies Targeting the Lymphatic Vasculature: Implications for Cancer Treatment

The potential benefits of anti-lymphangiogenic therapies for cancer treatment have been demonstrated in several pre-clinical cancer models, where blocking of VEGF-C and VEGF-D led to dramatic reduction in lymphatic vessel growth and metastasis formation (reviewed in Zheng et al., 2014). Moreover, several clinical studies targeting tyrosine kinase receptors including VEGFR1-3, FGFR, Tie2, C-MET and PDGFR-beta, that block both angiogenesis and lymphangiogenesis, reported increased survival of tumor bearing mice and human patients following treatment (reviewed in Duong et al., 2012; Dieterich and Detmar, 2016; Yamakawa et al., 2018).

During the past two decades several attempts have been made to block the VEGF-C/VEGFR3 axis at different levels of the signaling cascade. One way is designed to inhibit the VEGF-C/VEGFR3 axis by trapping the available VEGFR3 ligands- VEGF-C and VEGF-D-, before their interaction with the receptor. Several studies have pursued this option by generating soluble forms of VEGFR3 that can act as decoy receptors. For instance, tumor cells expressing soluble VEGFR3-Ig reduced tumor lymphatic formation and lymph node metastasis in mice (He et al., 2002) and rats (Krishnan et al., 2003). Moreover, soluble VEGFR3Ig suppressed both tumor size and tumor-associated lymphatic vessels in VEGF-C expressing tumors (Karpanen et al., 2001) and intravascular administration of soluble AdVEGFR3 IgG in mouse xenotransplants, inhibited also the formation of lymph node macrometastasis (He et al., 2005).

Another way to impede ligand-receptor interactions takes advantage of specific blocking antibodies. One such example is a monoclonal antibody neutralizing VEGF-D, which efficiently blocked lymph node metastasis in mice (Achen et al., 2000; Stacker et al., 2001). Similarly, systemic treatment with anti-VEGF3 antibodies specifically inhibited lymphangiogenesis and reduced lymphatic metastasis (Shimizu et al., 2004; Roberts et al., 2006). Ligand–receptor interactions were also blocked by VEGFR3 monoclonal antibodies (Persaud, 2004). This approach was shown to affect tumor growth and tumor angiogenesis (Kubo et al., 2000; Laakkonen et al., 2007; Tammela et al., 2008) and reduce intratumoral lymphatic formation (Laakkonen et al., 2007) in mouse xenograft models. In contrast, monotherapy using a humanized monoclonal anti-VEGFR3 (LY3022856) in phase 1 clinical trials, revealed no significant anti-tumor effect (Saif et al., 2016).

Finally, the VEGFR3 axis can be neutralized by preventing its downstream signaling. Tyrosine kinase inhibitors (TKIs) have been widely developed as downstream signaling blockers (reviewed by Tan et al., 2018; Qin et al., 2019). These small chemicals are extensively used for clinical and experimental purposes albeit being promiscuous since they block a variety of other kinases. Some of the current FDA-approved multi-kinase inhibitors are: Sorafenib (Ranieri et al., 2012), Sunitinib (Le Tourneau et al., 2007), Pazopanib (Harris et al., 2008), Axitinib, Regorafenib, Cabozantinib, Nintedanib, and Lenvatinib (Qin et al., 2019). Strikingly, the use of Sunitinib induced *Vegfc* expression and promoted lymphangiogenesis *in vivo.* Moreover, renal cell carcinoma patients treated with Sunitinib developed more lymph node metastasis in comparison with patients treated with other therapies (Dufies et al., 2017). Thus, TKIs specifically aimed at preventing lymphatic signaling are yet to be designed. Recently, SAR131675 – a novel VEGFR3-specific TKI – has shown promising results in the modulation of tumor and metastasis growth, by decreasing lymphatic vessel formation and macrophage infiltration *in vivo* in murine tumor and inflammation models (Alam et al., 2012; Hwang et al., 2019). To date, however, no VEGFR3-specific inhibitors have shown potential benefits for human use.

One question that remains open is whether manipulation of VEGF-C/VEGF-D targets only sprouting from pre-existing lymphatics, or affects also non-endothelial cells. The mechanisms underlying lymphatic differentiation from BMDCs and/or alternative progenitors, if any, are still poorly defined and may prove substantially different from that of ECs. Further characterization of the molecular pathways leading to lymphatic specification and integration of non-ECs into lymphatic vessels may provide alternative targets for the inhibition of tumor-induced lymphangiogenesis.

In addition to blocking the VEGF-C/VEGFR3 axis, the identified roles of the WNT and BMP pathways in LEC specification, may provide novel therapeutic opportunities. Notably, *Wnt5a* expression in gastric cancer was found to highly correlate with lymph node metastasis (Kurayoshi et al., 2006). While the proposed mechanism involved direct effects of WNT5A on tumor cell migration (Liu et al., 2013), it is possible that WNT5A acts also as a pro-lymphangiogenic factor in this setting, thereby increasing the incidence of lymph node metastasis. In similar fashion, COUP-TFII has also been associated with increased malignancy, lymph node metastasis, and poor prognosis. High levels of COUP-TFII expression were reported to correlate with aggressive behavior in patients with breast, pancreatic, and colon cancer (Qin et al., 2014), underscoring the potential benefits of blocking this nuclear receptor for cancer treatment. An alternative strategy for targeting lymphangiogenesis could be the use of agonists of BMP signaling, which is known to block this process in the developing embryo (Levet et al., 2013; Yoshimatsu et al., 2013; Dunworth et al., 2014; Jun-Dae and Jongmin, 2014; Cha et al., 2016). Recently, DNA decoys targeting the SOX-HMG family of transcription factors were shown to inhibit Sox18 activity and to block SOX18 interaction with *PROX1* DNA (Klaus et al., 2016), thereby presenting a unique alternative to target an otherwise undruggable protein.

The above-described treatments are mostly designed to disrupt the tumor-associated lymphatic vasculature in order to block tumor dissemination. Instead, new approaches focus on the idea of making lymphatic vessels more functional, with the ultimate goal of improving the anti-tumoral immune response, as well as immunotherapy delivery (Lund et al., 2016; Fankhauser et al., 2017; Song et al., 2020). In their study, Lund et al. (2016) showed that B16F10 melanoma tumors implanted in mice devoid of skin lymphatics, displayed reduced immune cell infiltration and inflammatory cytokines, suggesting that regional lymphatics are necessary for proper antitumoral and inflammatory response. *Chy* mice with defective lymphangiogenesis also mirrored the reduced immune cell recruitment phenotype in breast tumors. Interestingly, the expression of the lymphatic markers *LYVE1* and *PDPN* correlated as well with the immune response in a cohort of human metastatic cutaneous melanoma samples (Lund et al., 2016). In similar fashion, Fankhauser et al. (2017) showed that inhibition of lymphangiogenesis in a mouse melanoma model inhibited T cell recruitment and blocked the subsequent immune response. Moreover, they could confirm that *VEGFC* levels correlate with immunotherapy response in human patients (Fankhauser et al., 2017). Finally, both lymphatic vessel density and VEGF-C levels correlated also with the infiltration of CD8+ T cells in human primary melanoma and lymph node metastasis (Bordry et al., 2018).

The “re-discovery” of meningeal lymphatics, opens up new therapeutic opportunities for the treatment of rather inaccessible brain tumors. Recently, a new study has described that lymphatic-mediated immune functions in the brain were instrumental to enable a proper immune response against glioblastoma. Ectopic expression of VEGF-C agents increased CD8+ T cell drainage to the cervical lymph nodes, improving immune cell infiltration into the tumor mass. Furthermore, VEGF-C expression in combination with immunotherapy promoted tumor cell elimination and increased survival (Song et al., 2020). Finally, since immune cell infiltration density in tumors can serve as a predictive marker (Mlecnik et al., 2016), it would be interesting to investigate the correlation between tumor lymphatic formation and the potential success of immunotherapy. Altogether, these new lines of investigation present the lymphatics not as passive routes for metastatic cells toward the lymph nodes but rather as active players in the generation of anti-tumoral response.

## Future Prospects and Conclusion

The origin of lymphatic vessels in development and cancer has been a subject of intense debate. The proposed contribution of different endothelial and non-endothelial cells to pathological lymphangiogenesis indicates the great complexity underlying the initial stages of lymphatic vessel formation in tumors. Understanding the molecular mechanisms that induce LEC specification in different cell populations contributing to tumor-related lymphatic vessels, as well as those that propel growth and remodeling of pre-existing vessels, will most likely improve our ability to abrogate tumor lymphangiogenesis and promote the treatment of patients with metastatic disease.

## Author Contributions

KY and LG-M wrote, reviewed, and edited the manuscript. Both authors contributed to the article and approved the submitted version.

## Conflict of Interest

The authors declare that the research was conducted in the absence of any commercial or financial relationships that could be construed as a potential conflict of interest.
